# Modulation of allograft immune responses by *Porphyromonas gingivalis* lipopolysaccharide administration in a rat model of kidney transplantation

**DOI:** 10.1038/s41598-024-64771-5

**Published:** 2024-06-17

**Authors:** Yu Sato, Hiroshi Noguchi, Shinsuke Kubo, Keizo Kaku, Yasuhiro Okabe, Hideya Onishi, Masafumi Nakamura

**Affiliations:** 1 Department of Surgery and Oncology, Graduate School of Medical Sciences, Kyushu University, 3-1-1, Maidashi, Higashi-ku, Fukuoka, 812-8582 Japan; 2 Department of Cancer Therapy and Research, Graduate School of Medical Sciences, Kyushu University, 3-1-1, Maidashi, Higashi-ku, Fukuoka, 812-8582 Japan

**Keywords:** Kidney transplantation, Periodontitis, *Porphyromonas gingivalis*, Lipopolysaccharide, Endotoxin tolerance, Immunology, Nephrology

## Abstract

Periodontitis is a chronic inflammatory disease that affects the periodontal tissues. Although it is associated with various systemic diseases, the impact of periodontitis on kidney transplantation (KT) outcomes, particularly allograft rejection, remains unclear. This study investigated the effect of periodontitis on transplant immunity, specifically examining *Porphyromonas gingivalis*-derived lipopolysaccharide (LPS-PG). In vitro experiments revealed that LPS-PG increased regulatory T cells (Tregs) in Lewis rat spleen cells. In a mixed lymphocyte reaction assay, concentrations of interferon-γ, indicative of alloreactivity, were lower than in controls when LPS-PG was added to the culture and when LPS-PG-administered Lewis rat spleen cells were used as responders. In a rat KT model, LPS-PG administration to recipients promoted mild tubulitis and low serum creatinine and blood urea nitrogen levels 5 days post-KT compared with PBS-administered controls. Furthermore, LPS-PG-administered recipients had an elevated Treg proportion in their peripheral blood and spleen cells, and increased infiltrating Tregs in kidney allografts, compared with controls. The elevated Treg proportion in peripheral blood and spleen cells had a significant negative correlation with serum creatinine, suggesting elevated Tregs modulated allograft rejection. These findings suggest that periodontitis might modulate alloimmune reactivity through LPS-PG and Tregs, offering insights to refine immunosuppressive strategies for KT recipients.

## Introduction

Periodontitis is a prevalent chronic inflammatory disease that affects periodontal tissues, leading to the loss of periodontal attachment^1^. Despite being a localized inflammatory condition, periodontitis is associated with the exacerbation and promotion of various systemic diseases, such as chronic kidney disease^2,3^, atherosclerotic disease^4^, diabetes^5^, and others. Furthermore, periodontitis has the potential to induce systemic inflammation and impact host immune systems^6^. Most solid organ transplantation patients, including kidney transplantation (KT) recipients, are affected by periodontitis^7^. This condition might influence outcomes following KT, including allograft rejection.

However, there have been few previous reports of the association between periodontitis and KT, especially allograft rejection. The impact of periodontitis on kidney allograft rejection remains controversial. A previous study reported a positive correlation between acute rejection episodes after KT and periodontitis severity^8^. In contrast, another study reported that severe periodontitis was independently associated with a lower incidence of acute T cell-mediated rejection after KT, suggesting a potential effect of periodontitis on immune functions^9^.

We previously reported an experimental periodontitis model where mice were orally administered the periodontal pathogen *Porphyromonas gingivalis* (*Pg*), which prolonged allograft survival after skin transplantation through gut microbiota dysbiosis and the induction of regulatory T cells (Tregs)^10^. The proposed mechanism through which periodontal pathogens have a systemic influence involves an indirect process via microbiota dysbiosis and the direct impact of infectious agents and their products, including lipopolysaccharide (LPS)^11^. A previous study showed increased systemic LPS levels in periodontitis patients compared with healthy controls^12^. Although LPS mainly stimulates the innate immune system, the repeated challenge with LPS induced hyporesponsiveness in immune cells^13^. Additionally, LPS from *Pg* (LPS-PG), which is unique in terms of its chemical structure and receptor, also induces such hyporesponsiveness in immune cells^14,15^. On the basis of these previous studies, we hypothesized that LPS-PG could be a factor in how periodontitis affects transplant immunity; however, no prior reports exist on the effects of LPS-PG on transplant immunity, including acute rejection after KT.

Although the advancement of immunosuppression therapy has enhanced long-term graft survival after KT^16^, recipients often develop infections, drug toxicity, and other complications^17–19^. To prevent rejection and the adverse effects of immunosuppressive agents, strategies that consider individual factors for immunosuppression therapy are required. If periodontitis can influence transplant immunity, it should be considered when planning immunosuppression therapy for KT recipients. In this study, we assessed the impact of periodontitis on transplant immunity, with a specific focus on LPS-PG, using a rat model of KT.

## Results

### LPS-PG increases proportions of Treg and decreases alloreactivity in vitro

**Figure 1:**
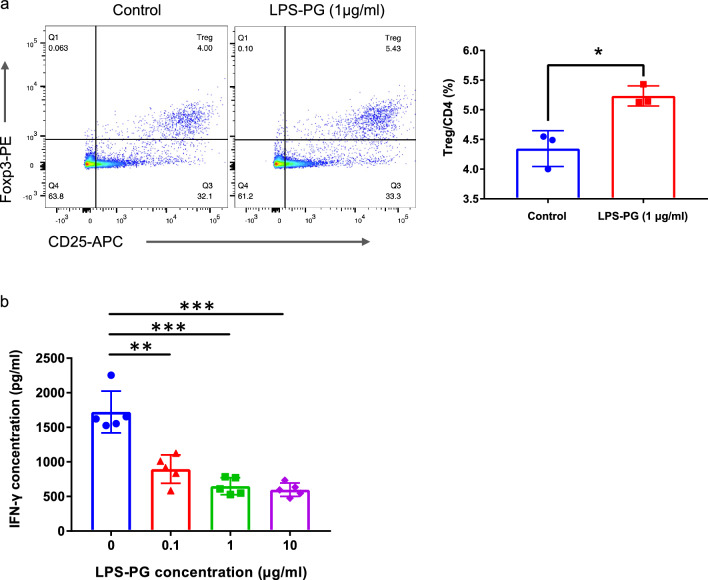
(**a**) Flow cytometry analysis of in vitro cultured LEW spleen cells with or without LPS-PG. The proportion of Tregs in total CD4 + T cells was determined. (**b**) IFN-γ concentrations after MLR assay with graded concentrations of LPS-PG were analyzed by ELISA. Statistical analysis was conducted to identify differences between groups. *p < 0.05, **p < 0.005, *p < 0.001.

On the basis of our previous findings, where the oral administration of *Pg* increased the proportion of Tregs in the spleens and peripheral blood of mice^10^, we examined whether LPS-PG could similarly induce the proportion of Tregs in Lewis (LEW) rat spleen cells in vitro (Fig. 1a). The proportion of CD4^+^ CD25^+^ Foxp3^+^ Tregs in CD4^+^ cells was higher after co-culture with LPS-PG compared with controls.

Subsequently, we assessed the impact of LPS-PG on alloreactivity using a mixed lymphocyte reaction (MLR) assay, using LEW rat spleen cells as responder cells and irradiated Brown Norway (BN) rat spleen cells as stimulator cells. LPS-PG promoted a dose-dependent significant reduction in IFN-γ production from responder cells (Fig. 1b).

### LPS-PG decreases alloreactivity in vivo

**Figure 2:**
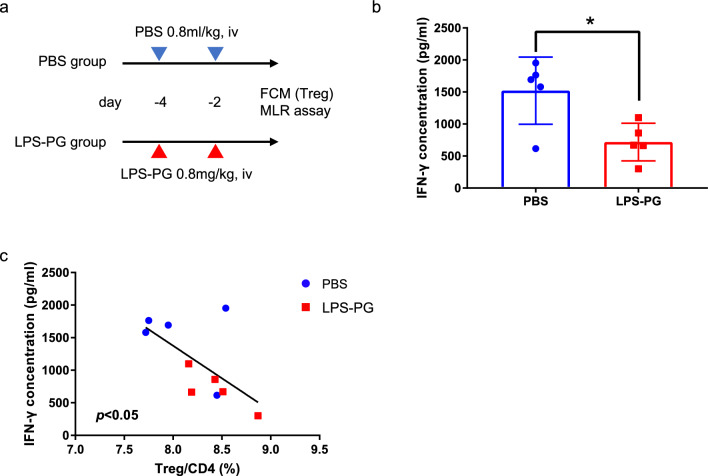
(**a**) Study design for the in vivo experiment. (**b**) IFN-γ concentrations after MLR assay using spleen cells from both groups as responder cells. (**c**) The association between IFN-γ concentration after MLR assay and Treg proportion before the MLR assay, determined by flow cytometry. Statistical analysis was conducted to identify differences between groups. *p < 0.05.

To assess the impact of LPS-PG in vivo, we divided LEW rats into LPS-PG and PBS groups. Rats in the LPS-PG group were administered LPS-PG intravenously twice and as a control, those in the PBS group were injected with the same volume of PBS. Two days after the injections, rats in both groups were sacrificed and spleen cells were collected (Fig. 2a). An MLR assay was conducted using the collected spleen cells as responder cells. IFN-γ was significantly decreased in the LPS-PG group (Fig. 2b). Furthermore, a significant negative correlation was observed between the Treg/CD4 proportion of spleen cells and IFN-γ production after the MLR assay (Fig. 2c).

### LPS-PG suppresses kidney allograft rejection after transplantation

**Figure 3:**
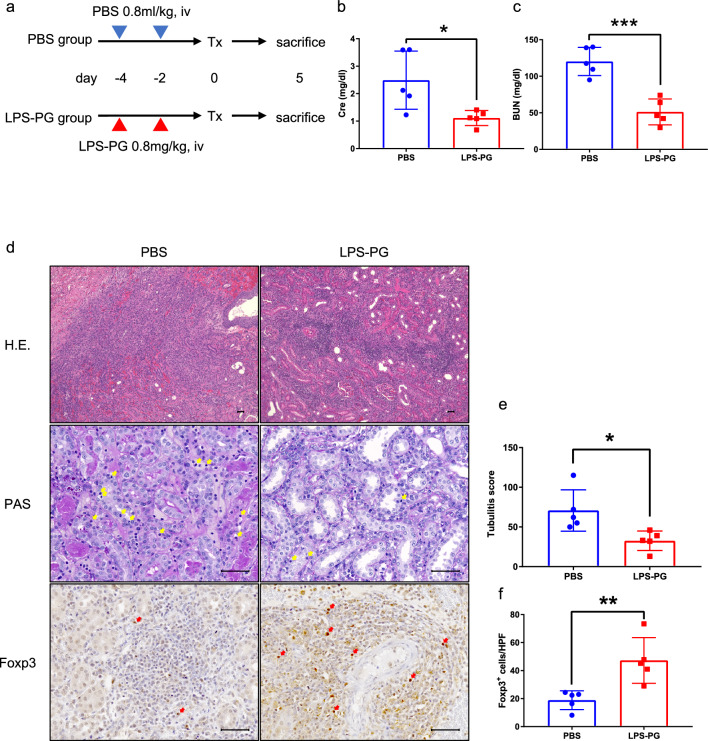
(**a**) Study design for the kidney transplantation experiment. (**b**) Serum creatinine and (**c**) blood urea nitrogen concentrations 5 days after transplantation. (**d**) Histology and immunohistochemistry of kidney allografts 5 days after transplantation. Yellow arrows indicate mononuclear cells infiltrating the tubule and red arrows indicate Foxp3 + cells. Scale bar = 50 μm. (**e**) The tubulitis score of each group was calculated as described in the Methods. (**f**) Calculated number of Foxp3 + cells in a high-power field using a digital image analysis program. Statistical analysis was conducted to identify differences between groups. *p < 0.05, **p < 0.005.

To assess the impact of LPS-PG on allograft rejection, we conducted orthotopic kidney transplantation following LPS-PG injection and sacrificed rats 5 days post-transplantation (Fig. 3a). Serum creatinine and blood urea nitrogen (BUN) levels 5 days post-transplantation were significantly lower in the LPS-PG group (Fig. 3b,c). The pathological findings of kidney allografts are shown in Fig. 3d. Although there was no significant difference in interstitial inflammation, the tubulitis scores of kidney allograft specimens from the LPS-PG group 5 days post-transplantation were significantly lower than those of the PBS group (Fig. 3e), suggesting that allograft rejection was suppressed in the LPS-PG group compared with the PBS group.

**Figure 4:**
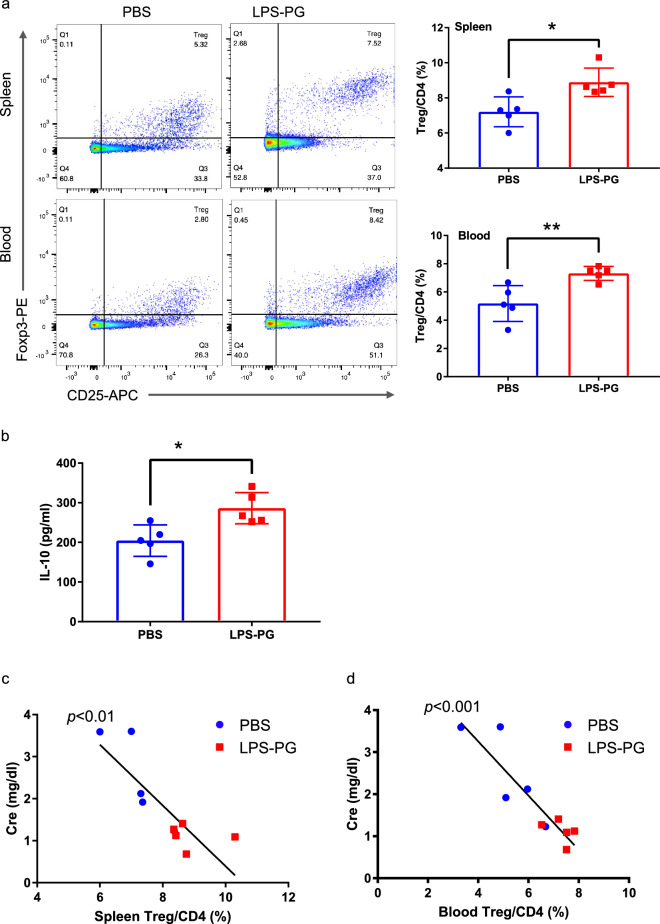
(**a**) Flow cytometry analysis of spleen cells and peripheral blood from both groups 5 days after transplantation. The proportion of Tregs in total CD4 + T cells was determined. (**b**) IL-10 concentrations in the serum 5 days after transplantation measured by ELISA. (**c**) The association of creatinine with spleen cells and (**d**) peripheral blood. Statistical analysis was conducted to identify differences between groups. *p < 0.05, **p < 0.005.

We also assessed the impact of Tregs on these results. The Treg/CD4 proportion in spleen cells and peripheral blood as well as the serum IL-10 concentrations were significantly higher in the LPS-PG group compared with the PBS group (Fig. 4a,b). Serum creatinine had a significant negative correlation with the Treg/CD4 proportion in the spleen and blood (Fig. 4c,d). Immunohistochemistry analysis demonstrated that the number of Foxp3^+^ cells in the kidney grafts was significantly higher in the LPS-PG group than in the PBS group (Fig. 3f).

## Discussion

In this study, we revealed the impact of LPS-PG on kidney allograft rejection. LPS-PG inhibited IFN-γ secretion in the MLR assay, indicating its ability to suppress T-cell responses to allogeneic donor stimulation. Subsequently, rats in the LPS-PG group had lower serum creatinine and BUN levels post-transplantation, along with a reduced tubulitis score in kidney allograft specimens, suggesting that LPS-PG suppressed allograft rejection. Furthermore, LPS-PG increased the population of Tregs in vitro and in vivo. The Treg/CD4 proportion had a significant negative association with the IFN-γ concentration in the MLR culture, as well as with serum creatinine levels after transplantation. This suggests that Tregs induced by LPS-PG suppressed alloreactivity in vitro and in vivo.

Although periodontitis is a localized inflammatory disease of the gingival tissue, it can trigger systemic inflammation and have an impact on the systemic immune system^6^. Various mechanisms have been proposed to explain how periodontitis induces systemic inflammation, including a direct effect by periodontal pathogens and an indirect effect through the dysbiosis of gut microbiota induced by swallowed periodontal pathogens such as *Pg*^11^. Our previous study reported the indirect effect of periodontitis whereby microbiota dysbiosis induced by the oral administration of *Pg* suppressed skin allograft rejection in a murine model^10^. In the current study, we extended our findings to demonstrate the direct effect of periodontitis, revealing that LPS-PG increased Tregs and suppressed kidney allograft rejection in rats.

LPS is an evolutionarily conserved pathogen-associated molecular pattern and LPS stimulation promoted the expression of proinflammatory cytokines through Toll-like receptors (TLRs) via pattern recognition^20,21^. Conversely, exposure to LPS renders immune cells unresponsive to subsequent challenges with LPS, a phenomenon known as endotoxin tolerance (ET)^22^. Although ET offers protective benefits against lethal bacterial infections by reducing the overproduction of proinflammatory cytokines, it can also render the host susceptible to infections due to its immunosuppressive effect^20^. A previous study reported that the induction of ET protected hosts from ischemia/reperfusion injury in the kidney and liver by inhibiting proinflammatory cytokines, including TNF-α, IL-6, and others^23–25^. Additionally, the impact of ET on organ transplantation has been explored in animal models. A previous report suggested that inducing ET in donor mice extended heart allograft survival in a murine heart transplantation model by promoting the generation of myeloid-related dendritic cells and Th2 responses^26^. Additionally, inducing ET in recipient rats delayed acute rejection in a hindlimb transplantation model by suppressing proinflammatory cytokines^27^. However, these previous studies used LPS derived from *E. coli* for ET induction. To the best of our knowledge, no previous study has reported the effect of ET induced by LPS-PG on organ transplantation, although it has been documented that LPS-PG induced ET in vitro similar to LPS from *E. coli*^15^. Local tissue destruction by periodontitis can lead to the systemic release of LPS, resulting in a high concentration of LPS in the peripheral blood of periodontitis patients^12,28^. Our data, indicating that LPS-PG suppresses kidney allograft rejection, suggests that ET induced by LPS-PG might explain the previously reported association between severe periodontitis and lower risk of T-cell-mediated rejection after KT^9^. However, what degree of periodontitis causes the sufficient secretion of LPS-PG to increase the proportion of Tregs in KT recipients has not been clarified. Further investigation into the association among clinical indices of periodontitis, serum LPS levels, and Treg proportions is needed in the future.

In this study, LPS-PG increased the Treg/CD4 proportion. The receptor for LPS-PG is TLR2 and the receptor for LPS derived from *E.coli* is TLR4^15^. Although TLRs are important receptors expressed on innate immune cells, TLR2 is functionally expressed on innate immune cells as well as activated T cells^29^. Furthermore, TLR2 signaling induced Treg proliferation by a MyD88-dependent mechanism in vitro^30^, suggesting that the increased Treg in our study may be attributed to TLR2 stimulation by LPS-PG. Our data also demonstrated an increase in Tregs in kidney allografts in the LPS-PG group rats. This might be a result of TLR2 stimulation by LPS-PG, as TLR2 stimulation promoted Treg migration^31^. Tregs are considered to be instrumental in determining the tolerance of an allograft^32^. Indeed, high levels of Foxp3-positive Tregs in peripheral blood and kidney allograft tissues after transplantation were associated with better graft survival^33–35^. In this study, elevated levels of Tregs were linked to the suppression of T-cell responses to alloantigens in the MLR assay and the inhibition of allograft rejection after KT, suggesting that the increase and migration of Tregs contributed to the suppression of transplant immunity. Tregs are thought to have a suppressive effect through contact-dependent and -independent mechanisms. The contact-dependent mechanism involves a direct effect on antigen-presenting cells through surface antigens such as cytotoxic T lymphocyte antigen-4 and programmed cell death 1, whereas the contact-independent mechanism involves a suppressive effect mediated via anti-inflammatory cytokines, including IL-10^32^. In this study, the presence of Tregs infiltrated into kidney allografts in the LPS-PG group might indicate a contact-dependent mechanism, and the elevated serum IL-10 concentration in the LPS-PG group might indicate a contact-independent mechanism.

This study suggests that periodontitis suppresses allograft rejection after KT, but it does not necessarily imply that periodontitis is beneficial for KT recipients, which we previously referred to as the “paradox of transplantation”^10^. Periodontitis patients have a higher risk for cardiovascular disease and increased mortality^36,37^. Indeed, KT recipients with severe chronic periodontitis also have a lower survival rate after transplantation compared with those without severe chronic periodontitis^9,38^. Our results might be valuable for the treatment of KT recipients with periodontitis, influencing decisions on immunosuppressive agents and post-transplant infectious disease management. For KT recipients with periodontitis, it may be necessary to consider lower doses of immunosuppressive agents. However, when attempting to develop an optimal immunosuppressive protocol for patients with periodontitis, we should elucidate how periodontitis affects transplant immunity under immunosuppressants. Thus, we are currently studying the association between clinical outcomes after kidney transplantation and periodontal status under immunosuppressive therapy. In addition, even if periodontitis suppresses acute rejection, there is no evidence that it induces graft-specific tolerance. Therefore, KT recipients with periodontitis might experience infections after transplantation more often than those without periodontitis. To prevent over-immunosuppression, oral care and, if needed, intensive periodontitis treatment before transplantation would be effective for outcomes after KT. Additionally, when considering a situation where periodontitis progresses after transplantation, longitudinal follow-up for periodontal conditions after KT will be important.

This study had several limitations. First, we were unable to elucidate the signal pathway through which LPS-PG increased the Treg proportion in this study, although we speculate that it may involve the TLR2-MyD88 signaling pathway, as mentioned earlier. However, no previous study has directly clarified the mechanism of Treg induction by LPS-PG. Thus, further investigations are needed to clarify this mechanism in the future. The second limitation was that we assessed kidney allograft rejection based solely on tubulitis, which reflects T-cell-mediated rejection, and we did not evaluate antibody-mediated rejection (AMR). AMR after KT poses a significant challenge due to poor graft survival and the absence of a gold-standard treatment^39,40^. Considering this, further investigations are needed to explore the impact of periodontitis on AMR after KT. Another limitation was that we could not replicate the systemic LPS concentration of periodontitis patients in the experimental animal model. The half-life of LPS in vivo is known to be very short, reportedly within 30 min^41^. The serum LPS concentration in periodontitis patients is higher than that in healthy controls, which is associated with the continuous supply of LPS from periodontal tissues. Thus, duplicating the systemic LPS concentration induced by periodontitis in an experimental animal model is challenging^12^. Nevertheless, this is the first study to investigate the effect of LPS-PG on kidney allograft rejection, providing insights into a potential mechanism by which periodontitis influences transplant immunity.

In conclusion, our findings indicate that LPS-PG suppresses allograft rejection after KT by increasing the proportion of Tregs.

## Methods

### Ethical statement

All experimental protocols received approval from the Animal Care and Use Committee of Kyushu University (Approval Number: A22-380). Rat handling and experimental procedures adhered to the Principles of Laboratory Animal Care and the ARRIVE guidelines.

### Animals and reagents

Eight-to-nine-week-old male LEW and BN rats were sourced from SLC Japan Inc. (Tokyo, Japan) for this study. The rats were maintained under specific pathogen-free conditions with unrestricted access to food and water.

Lipopolysaccharide from *Porphyromonas gingivalis* (LPS-PG, standard, 1 mg/ml) was procured from InvivoGen (CA, USA). LEW rats were randomly assigned to two groups: a phosphate-buffered saline (PBS) group and LPS-PG group. Five rats in each group were used for the in vivo MLR assay and kidney transplantation experiments. LPS-PG or PBS was administered to each rat via the tail vein. A dose of 0.8 mg/kg LPS-PG was administered to the LPS-PG group rats, determined according to a previous study on the effect of ET on transplant immunity^27^. Blood samples from the tail vein were collected and serum samples were stored at − 80 ℃ until analyzed. Serum creatinine and blood urea nitrogen (BUN) levels were measured by a commercial service (Kyudo Company, Saga, Japan).

### In vitro assay

Spleen cells (2 × 10^6^ cells/ml) from LEW rats were cultured in 6-well plates and divided into two groups (n = 3 per group). The control group was cultured in RPMI-1640 medium (FUJIFILM Wako Pure Chemical Corporation, Osaka, Japan) supplemented with 10% fetal bovine serum, 50 μM 2-mercaptoethanol (Thermo Fisher Scientific, MA, USA), 1% Penicillin–Streptomycin Solution, and 1% sodium pyruvate (both from FUJIFILM Wako Pure Chemical Corporation). The LPS-PG group was cultured in the same medium with the addition of 1 μg/ml of LPS-PG. After 72 h, the cultured cells were collected and analyzed.

### Mixed lymphocyte reaction culture

Spleen cells (2 × 10^6^ cells/ml) from LEW rats were co-cultured with 30 Gy irradiated spleen cells (2 × 10^6^ cells/ml) from BN rats in a 96-well flat-bottom microplate (200 μl/well; Nunclon Delta Surface, Thermo Fisher Scientific), for 3 days. LPS-PG was added to the culture at graded concentrations.

### Enzyme-linked immunosorbent assay (ELISA)

Cell-free supernatants obtained after the MLR assay were used for the measurement of IFN-γ and rat serum collected 5 days after kidney transplantation was used for the measurement of IL-10 concentrations. This was accomplished using a Quantikine ELISA kit (R&D Systems, MN, USA), following the manufacturer’s instructions.

### Flow cytometry

Hemolyzed peripheral blood samples and spleen cells were stained with PE/Cyanine7 anti-rat CD4 antibody (BioLegend, CA, USA), APC anti-rat CD25 (BioLegend), and PE anti-mouse/rat/human Foxp3 (BioLegend). For Foxp3 staining, samples were fixed and permeabilized using the Transcription Factor Buffer Set (BD Pharmingen, NJ, USA) following the manufacturer’s instructions. The stained cells were detected using a cell analyzer (FACS Verse, BD, NJ, USA) and analyzed with FlowJo software (BD).

### Kidney transplantation

Surgery was conducted under isoflurane inhalation anesthesia. Left kidneys from BN rats were orthotopically transplanted into the abdomen of LEW rats. The renal artery, vein, and ureter were anastomosed using 10–0 nylon. The native right kidney of recipient LEW rats was removed at the end of the procedure. Animals were sacrificed 5 days after transplantation, and the kidney grafts, spleens, and peripheral blood were harvested. Animals that died within 4 days after surgery were considered to have technical graft failure and were excluded from the study.

### Histology and immunohistochemistry

The kidney grafts were extracted, fixed with 4% paraformaldehyde, dehydrated, and embedded in paraffin. Subsequently, the specimens were sectioned into 4 μm slices. Periodic Acid-Schiff staining was performed to evaluate tubulitis using a previously described scoring system as follows^42^: 250 tubular cross-sections per animal were examined and categorized as (i) normal, (ii) mild tubulitis (infiltration of one mononuclear cell per tubular cross-section), (iii) moderate tubulitis (infiltration of two or three mononuclear cells per tubular cross-section with disruption of the basement membrane), or (iv) severe tubulitis (defined as ≥ four infiltrating mononuclear cells per tubular cross-section). A score for the degree of tubulitis was calculated for each animal, where each normal tubule received a score of 0, mild tubulitis was assigned a value of 1, and the number of tubules with moderate and severe tubulitis was multiplied by 2 or 3, respectively. The total tubulitis score for each animal was the sum of these figures.

Sections were stained for Foxp3 (NB100-39002, Novus Biologicals USA, CO, USA) and counterstained with hematoxylin to assess Foxp3 positive cell infiltration. Quantification was performed by scoring the number of positive cells in five high-power fields per animal using a digital image analysis program. The mean of these scores represented the score for each animal. The images were captured using a microscope (BZ-X810, KEYENCE, Osaka, Japan) and analyzed on the HALO Image Analysis Platform (Indica Labs, NM, USA).

### Data analysis

Data are expressed as the mean ± standard deviation, and the Student’s *t*-test was used to analyze continuous variables. A *p*-value < 0.05 was considered statistically significant. All statistical analyses were conducted using JMP Pro software (version 17, SAS Institute Inc., Cary, NC, USA) and GraphPad Prism 7.03.

## Data Availability

All data supporting the findings of this study are available within the paper.
